# The association between consideration of future consequences and food intake is mediated by food choice motives in a French adult population

**DOI:** 10.1017/S1368980023002501

**Published:** 2024-02-08

**Authors:** Marc Bénard, Margaux Robert, Caroline Méjean, Benjamin Allès, Emmanuelle Kesse-Guyot, Pauline Paolassini-Guesnier, France Bellisle, Fabrice Etilé, Gérard Reach, Serge Hercberg, Mathilde Touvier, Sandrine Péneau

**Affiliations:** 1 Sorbonne Paris Nord University, Inserm U1153, Inrae U1125, Cnam, Nutritional Epidemiology Research Team (EREN), Epidemiology and Statistics Research Center – University of Paris (CRESS), 74, rue Marcel Cachin, 93017 Bobigny, France; 2 MOISA, Univ Montpellier, INRAE, CIRAD, CIHEAM-IAMM, Montpellier SupAgro, Montpellier, France; 3 Paris School of Economics and INRAE, UMR1393 PjSE, 48 Boulevard Jourdan, Paris, France; 4 Department of Endocrinology, Diabetes and Metabolic Diseases, Avicenne Hospital, Bobigny, France; 5 Public Health Department, Avicenne Hospital, Bobigny, France

**Keywords:** Consideration of future consequences, Food Intake, Food choice motives, France, Adults

## Abstract

**Objectives:**

Consideration of future consequences (CFC) distinguishes individuals who adopt behaviours based on immediate needs and concerns from individuals who consider the future consequences of their behaviours. We aimed to assess the association between CFC and diet, and testing the mediating role of food choice motives on this relationship.

**Design:**

Individuals (aged ≥ 18 years) completed the CFC-12 questionnaire in 2014, at least three 24-h dietary records, and a food choice motive questionnaire. A multiple mediator analysis allowed to assess the mediating effect of food choice motives on the cross-sectional association between CFC and diet, adjusted for socio-demographic factors.

**Setting:**

Data from the NutriNet-Santé cohort study.

**Participants:**

27 330 participants.

**Results:**

CFC was associated with all food choice motives (*P* < 0·001), with the strongest positive associations for avoidance for environmental reasons, absence of contaminants and health motives and the strongest negative associations for innovation and convenience. Positive total effects were found between CFC and the consumption of healthy food groups (fruits and vegetables, whole-grain foods, legumes), and negative total effects for alcohol, meat and poultry and processed meat (*P* < 0·001). CFC was positively associated with diet quality (*P* < 0·001). Across food groups, major mediators of these relationships were higher health (8·4–32·6%), higher environmental (13·7–22·1 %) and lower innovation (7·3–25·1 %) concerns.

**Conclusions:**

CFC was associated with healthier dietary intake, essentially mediated by a greater motivation of future-oriented participants for self-centred and altruistic outcomes, including health and environment. Focusing on the awareness of future benefits in public health interventions might lead to healthier dietary behaviours.

Current western eating patterns, which are characterised by high dietary fat, salt and sugar and low fruit and vegetable consumption, can lead to undesirable impacts on health and environment^(1)^. To encourage healthy food choices, there is a need to better understand the determinants of consumer motives when purchasing and eating food. Psychological factors represent important determinants of food choice^(2)^. Consideration of future consequences (CFC) is a psychological construct that measures the extent to which people consider the potential consequences of their actions and the extent by which they are influenced by these potential consequences^(3)^. Individuals with higher CFC are therefore expected to adopt a future-oriented lifestyle that favours their distant goals and long-term concerns over immediate needs.

CFC is associated with health decision-making behaviours^(4)^, such as less smoking in undergraduate students^(5)^, more physical exercise in students^(5,6)^ and greater preventive behaviours including getting screened for diabetes in an adult population^(7)^. These data suggest that individual differences in CFC could lead to differences in food choice and dietary intake. CFC has been, for example, shown to be positively associated with self-perceived healthy eating behaviours in students^(4,6,8)^ or healthier food choices in a representative sample of the Norwegian population^(9)^. More specifically, CFC was negatively associated with intention to consume fast food^(10)^ or to display emotional eating behaviour^(11)^, while in students, CFC was positively associated with a lower alcohol consumption^(3)^. The addition of CFC to a Health Belief Model improves its prediction of healthy behaviour, as the predictive capacity increases compared with models without CFC, which may be explained by the fact that CFC is a significant determinant of healthy dietary behaviour^(12)^. In another study, no association was observed with saturated fat or fruit and vegetables intake, which may be due to a potential mediation by intention^(13)^. In our study, we hypothesise that adults with higher CFC would favour choice and consumption of healthier food groups compared with individuals who favour immediate ‘rewards’.

Food choices are influenced by various motives such as healthiness^(14)^, taste^(14,15)^ or price^(14)^. Environmental motives have been shown to play an increasing role in food choice decisions^(14)^ and therefore on dietary intake^(16)^. In turn, there are numerous factors influencing food choice motives such as biological, social and cultural factors^(2)^. In particular, individual differences of CFC could explain differences in food choice motives, but to our knowledge, there are no data available in the literature on this potential association. We hypothesised that specific food choice motives result from considering immediate (e.g. taste or price) or future (e.g. health or environment) consequences and that food choice motives could mediate the association between CFC and food intake.

The purposes of the present cross-sectional study were to assess the associations between CFC and dietary intakes (diet quality and food groups) and to evaluate the potential mediating roles of food motives, with a focus on sustainability, on these associations in a large sample of French adults.

## Methods

### Population

This study was conducted as part of the NutriNet-Santé study, which is a large ongoing web-based prospective cohort started in France in May 2009. The rationale, design and methods of the study have been described elsewhere^(17)^. The overall aim of this study is to explore the relationships between nutrition and health, as well as the determinants of eating behaviour and nutritional status. Eligible participants are adults (age ≥ 18 years) of a French population who have access to the Internet. Participants were recruited through a vast multimedia campaign (television, radio, national and regional newspapers, posters, Internet). At inclusion, participants have to complete several web-based questionnaires via a secure website (http://www.etude-nutrinet-sante.fr) to assess their diet, physical activity, anthropometric measures, lifestyle characteristics, socio-economic conditions and health status. Participants then complete this same set of questionnaires every year. Another set of optional questionnaires related to determinants of eating behaviours, nutritional status and specific health-related aspects are sent to every participant each month. In the present cross-sectional study, we included participants who had completed the CFC questionnaire, the food motives questionnaire and at least three dietary records.

### Data collection

#### Consideration of future consequences

CFC was assessed with the French version of the CFC-12 questionnaire^(18)^ completed from June to November 2014. The CFC-12 is a 12-item self-report questionnaire^(3)^ developed to measure the extent to which individuals consider distant *v*. immediate consequences of their behaviour. Each item is measured on a 5-point Likert scale ranging from ‘extremely uncharacteristic’ to ‘extremely characteristic’. An example of the items of the CFC-12 is as follows: *I consider how things might be in the future, and try to influence those things with my day-to-day behavior*. The total score is obtained by summing all the item ratings and by dividing the result by 12 for a final score ranging from 1 to 5 (higher scores indicating greater CFC). Good internal consistency was obtained in our sample with a Cronbach’s *α* of 0·79.

#### Assessment of food choice motives during purchase

Data regarding food choice motives were collected from July to December 2013 using a validated questionnaire to measure food choice motives including sustainability concerns at the time of purchase^(19)^. The questionnaire is based on sixty-three items with items focusing on food in general (thirty-two items) or on specific food groups (meat/fish/fruits and vegetables/dairy products) (thirty-one items). Questions are formulated as follows: ‘When I purchase food/meat/fish/fruits and vegetables/dairy products, I take into account…’. Participants are asked to rate their level of agreement on a 5-point Likert scale from ‘I strongly disagree’ to ‘I strongly agree’. Respondents can also indicate that they do not know. The underlying structure of the questionnaire was determined by exploratory factor analysis and then internally validated by confirmatory factor analysis^(20)^. Reliability was also assessed by internal consistency of selected dimensions and test–retest repeatability. Nine food choice dimensions were identified following the confirmatory factor analysis^(1)^: ethics and environment (18 items, e.g. production waste, impact on earth’s resources)^(2)^, local and traditional production (12 items, e.g. proximity of production, support for small-scale producers)^(3)^, taste (4 items)^(4)^, price (6 items)^(5)^; avoidance for environmental reasons (4 items, e.g. not buying meat for environmental reasons)^(6)^, health (6 items, e.g. health impact, nutritional composition)^(7)^; convenience (4 items, e.g. cooking convenience)^(8)^, innovation (4 items, e.g. original or innovative product, innovative fabrication/conservation process) and^(9)^ absence of contaminants (5 items, e.g. additives, exposure to chemicals). The ‘avoidance for environmental reasons’ dimension is different from the motivation ‘ethics and environment’ as it implies a radical commitment for conserving the environment. Since each dimension consisted of different number of items (from 4 to 18 items), all dimensions of food motives were transformed into values ranging from 0 (no concern) to 10 (strong concern) to standardise ratings.

#### Assessment of food groups consumption and diet quality

We selected participants who completed at least three 24-h dietary records between the 2 years preceding and the 2 years following the completion of the CFC-12 questionnaire. Participants are requested to complete two records during the week, and one during the week-end for a better representativeness. For each day, participants indicate food consumed and portions sizes. The 24-h dietary record is completed by using an interactive interface and is designed for self-administration on the Internet^(21)^. Participants report all foods and beverages consumed at breakfast, lunch, dinner and other eating occasions. They estimate the amounts eaten using standard measurements or using validated photographs^(19)^. These photographs represent > 250 foods (corresponding to 1,000 generic foods). Participants can choose between 7 portion sizes for most food products: 3 main portion sizes plus 2 intermediate and 2 extreme sizes. Nutrient intakes are estimated by using the published NutriNet-Santé food composition table including > 2,000 foods^(22)^. Mean daily food intake (in g/d) is weighted for the type of day of the week (weekday or weekend). Under reporters were identified by using the method proposed by Black^(23)^ and were then excluded. The use of the 24-h dietary record has been validated in the NutriNet-Santé cohort^(21)^. For the present study, we defined seventeen food groups: fruit and vegetables; seafood (e.g. fish and shellfish); meat and poultry; processed meat; eggs; dairy products (e.g. milk, yogurts with less than 12 % of added sugar, cheese); milk-based desserts (e.g. sweet yogurts, flan, cottage cheese, cream desserts); starchy foods; whole-grain foods; legumes; fats (oil, butter and margarines); sugary and fatty foods (e.g. cakes, chocolate, ice cream, pancakes); sugars and confectionery (e.g. honey, jelly, sugar, candy); fast food (e.g. pizzas, hamburgers, sandwiches, hot dogs); appetisers (e.g. crisps, salted biscuits); non-alcoholic beverages (excluding water) and alcoholic beverages.

The modified French National Nutrition and Health Program Guideline Score (mPNNS-GS), which is an a priori nutritional diet quality score reflecting the adherence to the French nutritional recommendations, was assessed. It is based on the PNNS-GS score^(24)^ but accounts for dietary component only, excluding the physical activity component. The score includes twelve components: eight refer to food serving recommendations (fruit and vegetables; starchy foods; whole-grain products; dairy products; meat, eggs and fish; seafood; vegetable fat; water *v*. soda) and four refer to moderation of nutrients or food (added fat; salt; sweets; and alcohol). Points are deducted for overconsumptions of salt and added sugars from sweetened foods, and when energy intake exceeds the energy requirement (as assessed by physical activity level and BMR calculated using Schofield equations^(25)^) by more than 5 %. The score has a maximum of 13·5 points, with a higher score indicating a better overall nutritional quality of the diet.

#### Socio-economic and demographic data

Potential confounders of the relationship between CFC, food choice motives and dietary data were collected based on the latest data to the date of completion of the CFC-12, which are provided yearly by the participants after their inclusion: age (years), sex (male, female), education level (primary, secondary, undergraduate and postgraduate), occupational status (unemployed, student, self-employed and farmer, employee and manual worker, intermediate profession, managerial staff and intellectual profession and retired) and monthly income per household unit. Monthly income per household unit was calculated using information about income and household composition. The number of people in the household was converted into a number of consumption units according to the Organisation for Economic Cooperation and Development equivalence scale: one consumption unit is attributed for the first adult in the household, 0·5 for other persons aged 14 or older and 0·3 for children under 14^(26)^. Categories of monthly income were defined as follows: < 1,200; 1,200–1,799; 1,800–2,299; 2,300–2,699; 2,700–3,699 and > 3,700 euros per household unit as well as ‘unwilling to answer’.

### Statistical analysis

A total of 51 394 participants of the NutriNet-Santé cohort study completed the CFC-12 questionnaire. Among them, 439 participants were excluded because they presented an acquiescence bias (agreeing or disagreeing to all questions without consideration of reversed items). From the 50 955 remaining participants, 27 396 participants completed the questionnaire on food choice motives and at least three dietary records to compose the final sample. Compared with excluded participants (the 23 559 participants who only completed the CFC-12 questionnaire), included participants were older, had a higher proportion of female, a higher proportion of individuals with university education and a lower BMI (*P* < 0·0001).

Relationships between CFC and individual characteristics were described with Pearson correlations for continuous variables and linear regressions for categorical variables. Multivariable multiple mediator analyses were performed using structural equation modelling to test the mediation of food choice motives on the relationship between CFC and dietary data. Multiple mediator analyses were used instead of several single mediator analyses because of correlations between food choice motives^(27)^.

Indirect effects were computed as products of overall effects of CFC on food choice motives by overall effects of food choice motives on dietary data. The estimation of mediation effects was based on the significance of the indirect effects with 99·9 % CI to take into account multiple testing^(28)^. CI were based on bias-corrected bootstrapping (*n* 10 000). Effect size for mediation was calculated as a ratio of the indirect effect to the total effect, multiplied by 100 (only when the total effect was significant). Positive effect sizes were interpreted as mediation and negative effect sizes were interpreted as suppression (or inconsistent mediation). Suppression effect appears in a mediation model when direct and indirect effects have opposite signs^(29)^. While it is assumed that a mediation effect reduces the relationship between the independent and dependent variables (by partially explaining this relationship), suppression effect increases the magnitude of this relationship.

All models were fit with the maximum likelihood estimator and were adjusted on age, sex, education level, occupational status, monthly income per household unit and energy intake (except when energy intake was the outcome). Analyses were not stratified on sex since the interactions between CFC and gender were non-significant considering total effects (*P* > 0·05 for all food choice motives, *P* > 0·05 for most food groups) (except fruits and vegetables, meat, poultry and processed meat, whole-grain foods, confectionery and alcoholic beverages). Since frequencies of missing data on confounders were very low (< 0·2 %) (Table 1), complete-case analyses were carried out. Statistical analyses were performed using the lavaan package version 0.5-22^(30)^ (R software).

**Table 1:** Individual characteristics of the 27 396 included participants (NutriNet-Santé study, 2014)

	*n* 27 396		Consideration of future consequences (CFC-12)		*P* ^*^
All			3·39	0·58^†^	
Age, years	51·9	14·3	−0·14^‡^	–0·15, –0·13	<0·001
Sex (%)					<0·001
Female	77·9		3·38	0·58	
Male	22·1		3·42	0·58	
Education level (%)					<0·001
Primary	2·2		3·06	0·55	
Secondary	28·3		3·21	0·57	
Undergraduate	31·8		3·39	0·56	
Postgraduate	37·7		3·54	0·56	
Missing data	0·1				
Occupational status (%)					<0·001
Unemployed	8·8		3·36	0·62	
Student	1·2		3·53	0·57	
Self-employed, farmer	1·7		3·45	0·60	
Employee, manual worker	13·7		3·31	0·57	
Intermediate professions	15·7		3·43	0·54	
Managerial staff, intellectual professions	22·8		3·55	0·56	
Retired	36·2		3·30	0·58	
Monthly income^§^ (%)					<0·001
<1,200€	9·0		3·33	0·60	
1,200–1,799€	20·4		3·35	0·58	
1,800–2,299€	15·8		3·35	0·57	
2,300–2,699€	10·8		3·39	0·58	
2,700–3,699€	18·3		3·47	0·57	
> 3,700€	13·6		3·50	0·58	
Unwilling to answer	12·0		3·31	0·57	
Missing data	0·2				

CFC, consideration of future consequences, score ranges from 1 to 5.

* *P*-value based on linear regressions for categorical variables or Fisher’s z transformation for continuous variables.

† Mean ± sd, all such value.

‡ Pearson correlations (95 % CI), all such values.

§ Monthly income represents the household income per month calculated per consumption unit (CU). The number of people of the household was converted into a number of CU according to a weighting system: one CU is attributed for the first adult in the household, 0·5 for other persons aged 14 or older and 0·3 for children under 14.

## Results

### Individual characteristics of the sample

Table 1 shows crude relationships between individual characteristics of the participants and CFC. CFC was negatively correlated with age (*P* < 0·001), and CFC scores were higher in male (*P* < 0·001), in participants with a higher level of education (*P* < 0·001), in participants with a managerial or intellectual profession or in students (*P* < 0·001) and in participants with a higher monthly income (*P* < 0·001).

### Food choice motives, diet quality, energy intake and food groups consumption

Descriptive characteristics of food choice motives, diet quality, energy intake and food groups consumption are presented in Table 2. Highest scores regarding food choice motives were found for taste, health, absence of contaminants, local and traditional production and price, whereas lowest scores were found for ethics and environment, and innovation. Avoidance for environmental reasons and convenience showed intermediary scores.

**Table 2:** Descriptive characteristics of food choice motives, diet quality, energy intake and food group consumption in 27 396 participants (NutriNet-Santé study, 2014)

	Mean	sd
Food choice motives score (*n* 27 396)^*^		
Avoidance for environmental reasons	5·65	2·02
Absence of contaminants	7·36	2·26
Health	7·46	1·76
Local and traditional production	7·37	1·74
Ethics and environment	2·71	2·11
Taste	8·77	1·37
Price	7·16	1·97
Convenience	5·31	2·53
Innovation	3·29	2·05
Diet quality (*n* 23 591)		
mPNNS-GS^†^	7·62	1·44
Energy intake (*n* 27 396)		
Energy intake, kcal/d	1909·28	408·75
Food group consumption (*n* 27 396)		
Fruit and vegetables, g/d	382·00	187·12
Seafood, g/d	37·33	29·67
Meat and poultry, g/d	72·48	40·63
Processed meat	18·17	17·31
Eggs, g/d	13·11	13·95
Dairy products, g/d	186·61	134·99
Milk-based desserts, g/d	32·63	38·48
Starchy foods, g/d	218·43	82·71
Whole-grain foods, g/d	37·88	41·97
Legumes, g/d	10·76	16·27
Fats, g/d	21·79	12·58
Sugary and fatty foods, g/d	75·54	49·96
Sugars and confectionery, g/d	29·69	25·30
Fast food, g/d	31·42	32·39
Appetisers, g/d	5·13	7·72
Non-alcoholic beverages, g/d	501·04	337·68
Alcoholic beverages, g/d	98·98	132·59

* Food choice motives score range from 0 (no concern) to 10 (strong concern).

† mPNNS-GS, modified French National Nutrition and Health Program Guideline Score, ranging from 0 (low quality) to 13.5 (high quality).

### Association between consideration of future consequences and food choice motives

Table 3 presents the results of the association between the CFC and the nine food choice motives with adjustments on confounders. CFC was significantly associated with all dimensions (*P* < 0·001). Positive associations were the highest with avoidance for environmental reasons, absence of contaminants and health, while they were more limited with local and traditional production, ethics and environment and taste. Negative associations were the highest with convenience and innovation and more limited with price.

**Table 3:** Association between consideration of future consequences and food choice motives in 27 396 participants (NutriNet-Santé study, 2014)

Food choice motive dimensions	Consideration of future consequences (CFC-12) Slope	95 % CI	*P* ^*^
Avoidance for environmental reasons	0·62	0·58, 0·66	<0·001
Absence of contaminants	0·61	0·57, 0·65	<0·001
Health	0·53	0·49, 0·56	<0·001
Local and traditional production	0·38	0·34, 0·41	<0·001
Ethics and environment	0·32	0·28, 0·36	<0·001
Taste	0·07	0·04, 0·10	<0·001
Price	−0·11	–0·15, –0·07	<0·001
Convenience	−0·36	–0·41, –0·31	<0·001
Innovation	−0·37	–0·41, –0·33	<0·001

CFC, consideration of future consequences, score ranges from 1 to 5.

* *P*-value based on linear regressions adjusted for gender, age, education level, occupational status, and monthly income per household unit and energy intake.

### Association between consideration of future consequences, diet quality and food groups consumption

Table 4 presents the results of the associations between CFC, diet quality (*β* (99 % CI) = 0·26 (0·21–0·31)) and food groups consumption. Considering total effects, CFC was positively associated with diet quality and with the consumption of fruits and vegetables, starchy foods, whole-grain foods, legumes, sugars and confectionery and non-alcoholic beverages, while negatively associated with the consumption of meat and poultry, processed meat, milk-based desserts, sugary and fatty foods, fast food, appetisers and alcoholic beverages. Overall, always considering total effects, CFC was not associated with energy intake and consumption of seafood, eggs, dairy products and fats. When considering the direct effects, the associations were relatively similar. Some differences were observed for the association between CFC and the consumption of dairy products and fats, which were significant (positive and negative association, respectively) while the associations between CFC and the consumptions of milk-based desserts, sugary and fatty foods, appetisers and non-alcoholic beverages were non-significant.

**Table 4:** Mediation of the association between consideration of future consequences, diet quality and food group consumption by the nine food choice motives in 27 396 participants (NutriNet-Santé study, 2014)

	Total effect		Direct effect		Indirect effect							
					Avoidance for environmental reasons		Absence of contaminants		Health		Local and traditional production	
	*β*-coefficient	99·9 % CI	*β*-coefficient	99·9 % CI	*β*-coefficient and % mediated	99·9 % CI	*β*-coefficient and % mediated	99·9 % CI	*β*-coefficient and % mediated	99·9 % CI	*β*-coefficient and % mediated	99·9 % CI
Dietary quality (mPNNS-GS^*^)	**0·26†**	**0·21, 0·31**	**0·12**	**0·07, 0·17**	**0·016**	**0·002, 0·031**	**0·017**	**0·005, 0·029**	**0·115**	**0·098, 0·132**	**−0·013**	**–0·024, –0·004**
					**6·3‡**		**6·7**		**44·2**		**−5·2**	
Energy intake, kcal/d	−7·57	–20·36, 5·66	0·98	–12·24, 14·49	−2·90	–6·95, 0·88	−0·54	–3·71, 2·57	**−7·26**	**–10·42, –4·22**	1·92	–0·61, 4·69
					–		–		–		–	
Fruits and vegetables, g/d	**36·77**	**30·20, 43·37**	**19·66**	**13·22, 25·80**	1·22	–0·66, 3·32	0·74	–0·78, 2·29	**11·87**	**10·07, 13·91**	−0·39	–1·64, 0·86
					3·3		2·0		**32·3**		−1·1	
Seafood, g/d	−0·82	–1·79, 0·18	−1·60	–2·58, –0·52	**−0·50**	**–0·80, –0·20**	**0·69**	**0·45, 0·97**	**1·11**	**0·87, 1·36**	−0·12	–0·33, 0·08
					–		–		–		–	
Meat and poultry, g/d	**−5·30**	**–6·73, –3·86**	**−2·65**	**–4·13, –1·17**	**−1·17**	**–1·62, –0·77**	−0·27	–0·62, 0·05	−0·01	–0·31, 0·29	**0·53**	**0·24, 0·81**
					**22·1**		5·1		0·2		**−9·9**	
Processed meat, g/d	**−1·87**	**–2·44, –1·26**	**−0·91**	**–1·50, –0·31**	**−0·26**	**–0·43, –0·08**	−0·13	–0·27, 0·02	**−0·61**	**–0·78, –0·45**	**0·29**	**0·17, 0·42**
					**13·7**		7·1		**32·6**		**−15·6**	
Eggs, g/d	0·19	–0·35, 0·71	−0·14	–0·69, 0·41	0·01	–0·14, 0·18	0·10	–0·02, 0·22	**0·13**	**0·03, 0·24**	−0·04	–0·13, 0·07
					–		–		–		–	
Dairy products, g/d	3·09	–1·75, 8·20	**5·37**	**0·10, 10·52**	−0·53	–2·00, 0·92	**−1·69**	**–2·84, –0·50**	**5·01**	**3·86, 6·23**	**−2·40**	**–3·53, –1·40**
					–		–		–		–	
Milk-based desserts, g/d	**−2·49**	**–3·93, –1·18**	−0·49	–1·99, 0·87	−0·39	–0·80, 0·06	**−0·39**	**–0·74, –0·07**	−0·21	–0·54, 0·07	−0·15	–0·43, 0·12
					15·5		**15·5**		8·5		6·0	
Starchy foods, g/d	**7·91**	**5·52, 10·50**	**4·29**	**1·71, 6·81**	**1·75**	**0·97, 2·52**	0·04	–0·54, 0·59	**0·66**	**0·13, 1·24**	0·14	–0·33, 0·67
					**22·1**		0·5		**8·4**		1·7	
Whole-grain foods, g/d	**6·68**	**5·18, 8·34**	**2·57**	**1·09, 4·15**	**1·06**	**0·63, 1·55**	0·27	–0·06, 0·60	**2·00**	**1·61, 2·40**	−0·27	–0·57, 0·01
					**15·9**		4·1		**29·9**		−4·0	
Legumes, g/d	**1·62**	**1·01, 2·24**	**0·87**	**0·26, 1·48**	**0·25**	**0·08, 0·45**	0·07	–0·08, 0·21	0·07	–0·07, 0·19	−0·02	–0·12, 0·10
					**15·4**		4·2		4·1		−1·0	
Fats, g/d	−0·36	–0·78, 0·05	**−0·51**	**–0·93, –0·08**	−0·10	–0·23, 0·03	**0·15**	**0·06, 0·26**	**−0·14**	**–0·23, –0·04**	**0·12**	**0·04, 0·21**
					–		–		–		–	
Sugary and fatty foods, g/d	**−1·94**	**–3·53, –0·41**	−0·16	–1·76, 1·50	**−0·32**	**–0·82, 0·18**	−0·15	–0·52, 0·22	**−0·83**	**–1·20, –0·48**	**−0·36**	**–0·69, –0·01**
					**16·4**		7·8		**42·7**		**18·7**	
Sugars and confectionery, g/d	**1·39**	**0·56, 2·25**	**1·15**	**0·30, 2·05**	0·03	–0·25, 0·30	−0·04	–0·23, 0·17	−0·08	–0·28, 0·10	**0·22**	**0·04, 0·40**
					2·2		−2·6		−5·5		**15·7**	
Fast food, g/d	**−2·88**	**–3·97, –1·82**	**−1·33**	**–2·42, –0·23**	−0·01	–0·36, 0·30	**−0·28**	**–0·56, –0·01**	**−0·72**	**–1·00, –0·47**	−0·16	–0·39, 0·07
					0·5		**9·6**		**25·1**		5·6	
Appetisers, g/d	**−0·35**	**–0·70, –0·06**	−0·21	–0·52, 0·07	0·03	–0·06, 0·12	−0·05	–0·12, 0·02	**−0·10**	**–0·16, –0·05**	−0·05	–0·12, 0·01
					−8·8		14·9		**30·1**		15·2	
Non-alcoholic beverages, g/d	**23·61**	**11·94, 35·36**	8·19	–4·47, 19·97	**6·87**	**3·28, 10·79**	0·94	–1·84, 3·70	**3·48**	**0·80, 6·25**	−1·46	–3·90, 0·92
					**29·1**		4·0		**14·7**		−6·2	
Alcoholic beverages, g/d	**−19·52**	**–25·07, –14·51**	**−18·23**	**–23·82, –13·25**	0·25	–1·11, 1·62	0·18	–0·88, 1·40	**−3·34**	**–4·47, –2·24**	**0·99**	**0·11, 1·94**
					−1·3		−0·9		**17·1**		**−5·1**	

	Indirect effect									
	Ethics and environment		Taste		Price		Convenience		Innovation	
	*β*-coefficient and % mediated	99·9 % CI	*β*-coefficient and % mediated	99·9 % CI	*β*-coefficient and % mediated	99·9 % CI	*β*-coefficient and % mediated	99·9 % CI	*β*-coefficient and % mediated	99·9 % CI
Dietary quality (mPNNS-GS^*^)	−0·001	–0·006, 0·004	**−0·006**	**–0·011, –0·002**	**0·004**	**0·001, 0·007**	−0·004	–0·009, 0·001	**0·010**	**0·004, 0·017**
	−0·3		**−2·3**		**1·4**		−1·5		**3·8**	
Energy intake, kcal/d	−1·15	–2·45, 0·24	**0·59**	**0·14, 1·43**	0·04	–0·45, 0·60	**−2·27**	**–3·75, –1·10**	**3·01**	**1·65, 4·77**
	–		–		–		–		–	
Fruits and vegetables, g/d	**1·06**	**0·47, 1·83**	**−0·98**	**–1·76, –0·26**	**0·54**	**0·16, 1·09**	0·16	–0·37, 0·77	**2·88**	**2·06, 3·87**
	**2·9**		**−2·7**		**1·5**		0·4		**7·8**	
Seafood, g/d	**−0·22**	**–0·35, –0·11**	−0·01	–0·05, 0·03	0·03	–0·01, 0·08	**0·11**	**0·02, 0·21**	**−0·30**	**–0·44, –0·18**
	–		–		–		–		–	
Meat and poultry, g/d	**−0·98**	**–1·27, –0·71**	**0·15**	**0·04, 0·28**	**−0·18**	**–0·32, –0·06**	0·09	–0·03, 0·23	**−0·80**	**–1·03, –0·59**
	**18·5**		**−2·8**		**3·4**		−1·8		**15·2**	
Processed meat, g/d	**−0·19**	**–0·28, –0·12**	**0·06**	**0·02, 0·12**	**−0·06**	**–0·11, –0·02**	**0·07**	**0·02, 0·14**	**−0·14**	**–0·22, –0·07**
	**10·2**		**−3·3**		**3·3**		**−4·0**		**7·3**	
Eggs, g/d	0·04	–0·01, 0·09	−0·01	–0·03, 0·01	0·00	–0·01, 0·03	0·01	–0·04, 0·06	**0·07**	**0·01, 0·13**
	–		–		–		–		–	
Dairy products, g/d	**−1·51**	**–2·19, –0·99**	0·05	–0·12, 0·24	**−0·37**	**–0·74, –0·12**	**−0·46**	**–0·93, –0·05**	−0·38	–0·96, 0·11
	–		–		–		–		–	
Milk-based desserts, g/d	−0·13	–0·27, 0·01	**0·09**	**0·03, 0·18**	−0·04	–0·11, 0·01	**−0·17**	**–0·30, –0·05**	**−0·63**	**–0·84, –0·43**
	5·0		**−3·5**		1·5		**6·7**		**25·1**	
Starchy foods, g/d	**0·47**	**0·22, 0·78**	**−0·23**	**–0·47, –0·06**	−0·05	–0·18, 0·03	−0·12	–0·34, 0·09	**0·97**	**0·66, 1·33**
	**6·0**		**−2·9**		−0·7		−1·5		**12·2**	
Whole-grain foods, g/d	**0·53**	**0·34, 0·76**	**−0·15**	**–0·28, –0·04**	**0·20**	**0·06, 0·38**	−0·06	–0·21, 0·07	**0·54**	**0·36, 0·75**
	**7·9**		**−2·2**		**3·0**		−0·9		**8·1**	
Legumes, g/d	**0·17**	**0·10, 0·26**	**−0·06**	**–0·12, –0·02**	0·02	0·00, 0·06	0·03	–0·03, 0·09	**0·22**	**0·15, 0·31**
	**10·6**		**−3·8**		1·3		1·9		**13·7**	
Fats, g/d	0·03	–0·01, 0·07	0·01	0·00, 0·04	0·01	0·00, 0·04	**0·06**	**0·02, 0·10**	0·00	–0·05, 0·05
	–		–		–		–		–	
Sugary and fatty foods, g/d	0·14	0·00, 0·31	**0·07**	**0·01, 0·15**	−0·02	–0·10, 0·03	−0·13	–0·29, 0·01	−0·17	–0·37, 0·00
	−7·5		**−3·4**		1·2		6·9		8·9	
Sugars and confectionery, g/d	0·03	–0·05, 0·12	−0·01	–0·06, 0·02	0·02	–0·01, 0·07	−0·08	–0·16, 0·00	**0·14**	**0·04, 0·24**
	2·4		−1·0		1·3		−5·5		**10·0**	
Fast food, g/d	−0·03	–0·14, 0·08	**0·05**	**0·01, 0·12**	**−0·07**	**–0·14, –0·02**	−0·04	–0·14, 0·05	**−0·29**	**–0·44, –0·16**
	1·0		**−1·7**		**2·3**		1·5		**10·0**	
Appetisers, g/d	0·03	0·00, 0·06	0·00	0·00, 0·02	0·00	–0·01, 0·01	0·02	0·00, 0·05	−0·01	–0·05, 0·02
	−9·4		−1·1		0·5		−6·0		2·8	
Non-alcoholic beverages, g/d	0·37	–0·79, 1·59	−0·18	–0·75, 0·22	0·00	–0·46, 0·45	**1·49**	**0·41, 2·68**	**3·90**	**2·52, 5·53**
	1·6		−0·8		0·0		**6·3**		**16·5**	
Alcoholic beverages, g/d	**−0·47**	**–0·91, –0·07**	**0·33**	**0·10, 0·65**	0·03	–0·14, 0·21	**1·20**	**0·77, 1·74**	−0·47	–0·99, 0·01
	**2·4**		**−1·7**		−0·2		**−6·2**		2·4	

Significant effects are shown in bold (*P* < 0·001).

* mPNNS-GS, modified French National Nutrition and Health Program Guideline Score, analysis on 23 591 participants.

† *β*-coefficient (all such values): All estimates correspond to a 1-point increase of CFC (continuous variable ranging from 1 to 5) and are adjusted for gender, age, education level, occupational status, monthly income per household unit and energy intake (except when energy intake was the outcome).

‡ Percentage mediated (all such values): percentage mediated is the ratio of indirect effect on total effect and is not calculated when the total effect is non-significant. Positive percentages indicate mediation effect, whereas negative percentages indicate suppression effect (indirect and total effects have opposite signs).

### Mediation effects of food choice motives on the association between consideration of future consequences, diet quality and food groups consumption

Table 4 also presents the results of the mediation effects of food choice motives on the relationship between CFC, diet quality and food groups consumption (indirect effect). All food choice motives either mediated or suppressed (inconsistent mediation) the positive relationship between CFC and diet quality, apart from ethics and environment, and convenience. Health was the mediator with the strongest effect (44·2% of the relationship between CFC and diet quality was mediated by health, compared with less than 10% for the other mediators/suppressors). Overall, higher concerns for motivations such as health (from 8·4 % to 32·6 %), ethics and environment (from 2·4 % to 18·5 %) and avoidance for environmental reasons (from 13·7 % to 22·1 %), as well as lower concerns for innovation (from 7·3 % to 25·1 %) were consistent mediators of the relationship between CFC and food group consumption. Taste appeared to be a consistent suppressor of this relationship. The mediation or suppression effects of other food choice motives were more inconsistent across food groups. Absence of contaminants was the motivation with the least significant indirect effects.

## Discussion

In the present study, CFC was associated with better nutritional quality of the diet, reflected by higher intakes of food groups such as fruits and vegetables, whole-grain foods and legumes, and lower intakes of food groups such as alcoholic beverages, meat and poultry and processed meat. Overall, the main mediators of these relationships were higher concerns for health and the environment (avoidance for environmental reasons and ethics and environment), as well as lower concerns for innovation, whereas taste was the main suppressor.

### Consideration of future consequences and socio-economic and demographic data

Our findings that younger people were more future-oriented are consistent with previous findings. Older people have an increased risk of death, which may result in a shorter time horizon^(31)^. Our results also support previous data relating lower socio-economic status and higher immediate orientation, which can be due to perception of scarce control over future outcomes which is in fact a contextually appropriate response to the environment rather than a lack of willpower^(32)^. We also showed greater CFC scores in male compared with female. Contradictory results on sex have been reported in the literature, either with no association^(33)^ or with male having higher CFC scores than female^(34)^.

### Consideration of future consequences and food choice motives

Our results showed that individuals with a high consideration for the future were more concerned about the environment including avoidance for environmental reasons, and ethics and environment dimensions when purchasing food. They also had higher concerns for their health, as reflected by higher association with health and absence of contaminants dimensions. These future-oriented participants were also more interested in local and traditional production and, to a lesser extent, with taste compared with individuals with a lower CFC. Future-oriented individuals were less interested in convenience, innovation and price of the food products. In agreement with these observations, CFC was found associated with attitudes towards conservation and protection of the environment and the utilisation of natural resources^(35)^. CFC also showed associations with ethical decision-making, such as displaying stronger moral reasoning^(36)^ and more ethical negotiations strategies^(37)^. In addition, CFC has been positively associated with healthier food choice^(9)^ and consumers’ valuation for healthy labels or claims^(38)^ suggesting a greater interest for health motives, while specifically considering future health effects has been shown to improve the nutritional quality of choices and the likelihood of using nutrition information^(39)^. All these behaviours are characteristics of long-term goals^(4)^. CFC was also found associated with financial decision-making such as higher level of savings^(40)^. The negative association between CFC and price observed in the present study could be the result of a lack of perceived intertemporal dilemma regarding food financial decisions. Our results suggest that future-oriented individuals would tend to make food choices according to potential future outcomes of their behaviour, whether this behaviour leads to more self-centred outcomes (e.g. oriented towards better health) or more altruistic outcomes (e.g. oriented towards the environment).

### Consideration of future consequences, diet quality and food groups consumption

The present analysis showed that future-oriented individuals had a healthier diet overall compared with less future-oriented individuals. More specifically, higher CFC was associated with higher intakes of healthy food groups such as fruits and vegetables, starchy foods, whole-grain foods and legumes and negatively associated with unhealthy food groups such as meat and poultry, processed meat, milk-based desserts, sugary and fatty foods, fast food, appetisers and alcoholic beverages. However, CFC was also positively associated with some unhealthy food groups such as sugars and confectionery, and non-alcoholic beverages. Other studies have supported healthier behaviour in individuals with higher CFC, as they were more likely to report self-perceived healthy eating behaviours^(5–8,35)^, less likely to intend to consume fast food^(10)^ and less likely to drink alcohol^(3,5)^ compared with individuals with lower CFC. Studies based on relatively small samples of individuals showed no association between CFC and intake of fruit and vegetables or saturated fat^(13)^. Interestingly, while a study showed no association between CFC and self-perceived healthy eating, a significant association was observed when a food-specific version of the CFC was considered^(41)^. Finally, another study suggested that both CFC-Future and CFC-Immediate were significant predictor of consumption frequency of functional foods, that is, food product enriched with minerals, vitamins, fatty acids or proteins for health enhancement or disease prevention^(42)^. Exploring the mediating role of food choice motives allows us to propose explanations for these relationships between CFC and better overall diet quality.

### Mediating role of food choice motives

Health motive was the main mediator of the associations between CFC and intakes of fruits and vegetables, processed meat, whole-grain foods, sugary and fatty foods, fast food, appetisers and alcoholic beverages, which suggests that individuals with higher CFC are more likely to attempt complying with nutritional health guidelines than individuals with lower CFC. In France, nutritional guidelines recommended at the time of the study a greater consumption of fruit and vegetables, whole-grain foods and a lower consumption of milk-based desserts (high in sugar), sugary and fatty foods, alcohol, processed meat, fast food, and appetisers^(43)^.

Environmental concerns (avoidance for environmental reasons and ethics and environment) were also significant mediators of the association between CFC and consumptions of several food groups such as seafood, meat and poultry and processed meat. Reduction of meat, poultry and processed meat consumption would lead to lower nitrogen emissions^(44)^ and lower greenhouse emissions^(44)^ which is likely to encourage individuals with greater future orientation to decrease their intake of such products. Vegetarianism is characterised by no or low consumption of these foods for reasons related to animals’ health and ethical treatment, as well as the environmental protection^(45)^. Finally, environmental motivations are linked to higher organic food consumptions^(14)^, which were previously found to be associated with CFC^(46)^.

Innovation in food products was another consistent mediator. Since CFC is negatively associated with innovation, these results suggest that the lack of interest of future-oriented individuals towards innovation, which can be seen as a weak response to food marketing, contributes extensively to the differences in intakes of these food groups. Moreover, taste, which can be considered an immediate reward of food intake, was a consistent suppressor although the effect sizes were small. Motivation for taste suppressed higher diet quality, as well as higher intakes of fruits and vegetables, starchy foods, whole-grain foods, legumes and lower intakes of meat and poultry, processed meat, milk-based desserts, sugary and fatty foods, fast food and alcoholic beverages of individuals with higher CFC. These results support previous data suggesting that higher taste preferences are linked with a poorer diet quality^(15)^. Finally, weak mediations effects were found for price, which is consistent with the weak relationship observed between CFC and price in our sample.

Several motivations were involved simultaneously in most of the associations, showing a complex relationship between all these factors and arguing for better consideration in future works. For example, in the case of seafood, a negative indirect effect was found for environmental concerns while a positive indirect effect was found for health concerns. These indirect effects with opposite signs could potentially explain the non-significant total and direct effects between CFC and seafood, suggesting potential dilemmas in individuals regarding food choices, as previously reported^(47)^. Conflicting health and environmental recommendations exist for some food groups, as a higher nutritional quality is not always associated with a lower environmental impact^(48)^.

### Strengths and limitations

The main strength of this study is the use of three 24-h dietary records twice a year over a 4-year period, which allowed us to have a good representation of our participants’ food consumption. However, this method also has limitations such as being time consuming, showing desirability bias (survey-related dietary changes) and reported food consumption not always representative^(49)^. Another strength is the large study sample size with individuals of various socio-demographic characteristics and nutritional status, which allows the use of multiple covariates to adjust for confounding factors. However, we cannot rule out the possibility that other important confounders were not considered, either for the exposure–outcome relationship, the exposure–treatment relationship or the mediator–outcome relationship. The multiple mediator analysis allowed us to differentiate individual indirect effects from the multiple mediators, but the structural equation modelling approach cannot deal with interaction effects effectively. Another limitation of our study is its cross-sectional design with lack of temporal ordering in the mediation model, which, with other limitations, does not allow us to infer valid causal interpretations. Our study could also present a selection bias because of the method used to recruit participants, which was based on volunteering. Consequently, our participants were more often female and had a higher education, higher income and professional status^(50)^ and may have higher health awareness than the global population. Therefore, cautious is needed when extrapolating our result to the general population. Other strengths of the present study consist in the validated questionnaire used. The CFC-12 questionnaire has been widely used with health and environmental outcomes, while the food choice motive questionnaire includes motives rarely described in the literature in particular: avoidance for environmental reasons, local and traditional production and innovation dimensions.

### Conclusions

Our results showed that CFC was associated with healthier dietary intake such as higher intakes of fruits and vegetables, whole-grain foods, legumes and lower intakes of alcohol, meat and poultry and processed foods. Major mediators of these relationships were a greater motivation of future-oriented participants for both self-centred and altruistic outcomes, including health and environment, but also a lower motivation for innovation. Intervention studies are needed to understand how individual CFC level can be influenced and whether increasing awareness of future benefits could lead to healthier food choices and dietary behaviours.
